# Survey of real-time brainmedia in artistic exploration

**DOI:** 10.1186/s42492-024-00179-2

**Published:** 2024-11-18

**Authors:** Rem RunGu Lin, Kang Zhang

**Affiliations:** Computational Media and Art, Hong Kong University of Science and Technology (Guangzhou), Guangzhou 510000, Guangdong China

**Keywords:** Brain art, Brainmedia, interactive art, Electroencephalography, Neurofeedback, Artistic brain-computer interface

## Abstract

This survey examines the evolution and impact of real-time brainmedia on artistic exploration, contextualizing developments within a historical framework. To enhance knowledge on the entanglement between the brain, mind, and body in an increasingly mediated world, this work defines a clear scope at the intersection of bio art and interactive art, concentrating on real-time brainmedia artworks developed in the 21st century. It proposes a set of criteria and a taxonomy based on historical notions, interaction dynamics, and media art representations. The goal is to provide a comprehensive overview of real-time brainmedia, setting the stage for future explorations of new paradigms in communication between humans, machines, and the environment.

## Introduction

In contemporary media art, the integration of the mind, brain, and body with technological advancements blurs the boundaries between the physical and virtual as well as the organic and technological. This survey is positioned at this intersection, aiming to lay the groundwork for utilizing real-time brain signals in artistic exploration and to shed light on the potential for new forms of communication among humans, bodies, brains, and machines [1].

The human brain, often referred to as “the most marvelous machine in the known universe” [2] has captivated scientists, researchers, and artists for centuries. The development of brain-computer interfaces (BCIs), such as electroencephalography (EEG), started around 1930 and has expanded the possibilities for artistic expression since the 1960s [3]. Early BCI musical performance was pioneered by Alvin Lucier in 1965 [4], and subsequently conducted by Richard Teitelbaum [5] and David Rosenboom [6]. The term BCI itself was first introduced by Jacques Vidal in the 1970s to describe systems that detect brain activity for one-way communication with a computer [7]. Through these early experiments, real-time EEG feedback developed—from using brain-controlled machines to exploring the inner self and later, to envisioning the self as “connected with—or plugged into—broader circuits or systems” [3]. This progression highlights the social potential of computing, such as “augmenting the self,” “self-insight,” and “connecting with others” as described by Flora Lysen [3]. Flora Lysen called these envisioned BCI artworks ‘brainmedia’ [3], a term used throughout this survey. Brainmedia emphasizes how socio-technological practices, along with cultural imaginaries and visions, foster new forms of expression and understanding in areas such as synchronicity, communication, wholeness, control, augmentation, and awareness [3].

Prior to the 21st century, artistic applications of BCI were sparse and rudimentary. Advances in technology, particularly the availability of affordable BCIs and software development kits in consumer markets, have inspired artists from various disciplines to integrate neural activity into their artwork [1]. In recent years, BCI technology has also entered the mainstream of human-computer interaction, supported by advances in signal processing, machine learning, and visualization technologies [8].

This survey draws on the historical context to focus on artworks that utilize real-time brain signals for artistic exploration. By examining these developments, the aim is to provide a comprehensive overview of real-time brainmedia in the 21st century.

### Motivation

Recent BCI surveys primarily explored various technical aspects, including techniques, advancements, challenges, opportunities, and field applications [9–14]. Some studies reviewed the historical development and technological advancements of BCI systems [15] as well as the role of BCI in artistic contexts during its formative years from 1964 to 1977 [3].

In the realm of artistic BCI, significant contributions can be found in Anton Nijholt’s “Brain Art: Brain-Computer Interfaces for Artistic Expression,” which includes foundational texts on the introduction of BCIs for artistic expression [1] and a comprehensive survey with a taxonomy of BCI in contemporary art [16]. Prpa and Pasquier [16] categorized BCI artworks based on their inputs, mapping, and outputs, offering a nuanced framework for understanding these intersections. However, the focus remains predominantly on the technical aspects, output formats, and presentation styles of the artwork.

The history of brainmedia shows that artworks are often ahead of their time. Developed under the influence of cybernetic and biological theories, media ecological concepts, and countercultural movements [3], early brainmedia artworks of the 1960s and the 1970s laid the foundational ideologies that continue to influence current brainmedia. Delving into the theories of media ecology [17], countercultural movements [18], and cybernetics [19] facilitates a better understanding of contemporary artwork and helps address critical questions in contemporary media art. These questions include what new methods and inquiries are necessary as the entanglement between mind, brain, and body deepens, and as the boundaries between the physical and virtual or organic and technological become increasingly blurred.

By bridging the gaps between technical advancements, artistic expression, and cultural context, we can gain a deeper understanding of contemporary brainmedia and the questions it addresses today.

## Concept and scope

### Interactive art

Interactive art, commonly associated with digital art today, is difficult to define. Although the roots of interactive art can be traced back to the 1950s, it gained significant prominence when introduced as a distinct category at the Prix Ars Electronica in 1990 [20]. This event was also celebrated as “The Beginning of a New Art Form” by Roger F Malina [21]. Interactivity in art can occur between humans or between humans and non-human entities such as computer systems, inanimate objects, animals, and plants. Dinkla [22] defined interactive art as computer-supported works, emphasizing the necessary role of technology in facilitating interactive experiences. Furthermore, interactive art demands active engagement from participants, extending beyond mere mental perception [20].

The scope of interactive art has been challenged as the interactivity within an artwork has become more hybridized. Examples include artworks that incorporate internal system interactions with a data source but do not directly interact with their audiences [21]. In 2007, the Prix Ars Electronica introduced a new category labeled “Hybrid Art” for artworks that cannot be strictly classified as interactive art. The jury later noted that specific interest in this category shifted beyond information technologies to the realm of bio art [21]. By expanding the scope of interactive art to intersect with bio art, it becomes apparent that real-time brainmedia falls within this intersection. Real-time brainmedia offers a broader range of interactive approaches beyond traditional interactive art, which is elaborated on in a later chapter of this paper.

### Bio art

Bio art, introduced by Eduardo Kac in 1997 [23], is an emerging genre at the intersection of biology and artistic practices. It incorporates living biological materials, such as organisms and biodata, as well as artificial life, manipulating and modifying life processes and ecosystems to explore and challenge the ethical, social, cultural, and political boundaries between humans, non-humans, and the environment [24]. Researchers such as Eduardo Kac [25] and William Myers [26] have extensively explored these themes. Furthermore, as envisioned by Neri Oxman, this field represents a shift from nature-inspired designs to design-inspired nature [27]. Recently, Patrick McMillen and Michael Levin proposed a “unifying concept for integrating biology across scales and substrates,” which they termed “collective intelligence” [28], resonating with the expansive notion of ecology from the 1970s [29]. These works emphasize the manipulation of synthetic biology and the exploration of both human and artificial biology themes, demonstrating the convergence of biotechnology and art. Inspired by previous works on bio art, bio art is broadly categorized here into three subfields: natural biology, artificial biology, and human biology, each with a distinct focus and artistic approach.

Natural biology in bio art often involves the use of plants, animals, microorganisms, and natural data to explore and raise awareness of ecological and biological processes, benefiting from the availability of increasingly diverse data [30]. These artworks address themes such as environmental impacts, symbiotic relationships, and the ethical implications of human interactions with nature.

Artificial biology examines the creation and manipulation of synthetic biological systems. Artists in this domain utilize synthetic biology, robotics, and computational methods to create new life forms or biological systems that do not exist in nature, investigating “life-as-we-know-it” as well as “life-as-it-might-be” [31].

**Fig. 1:**
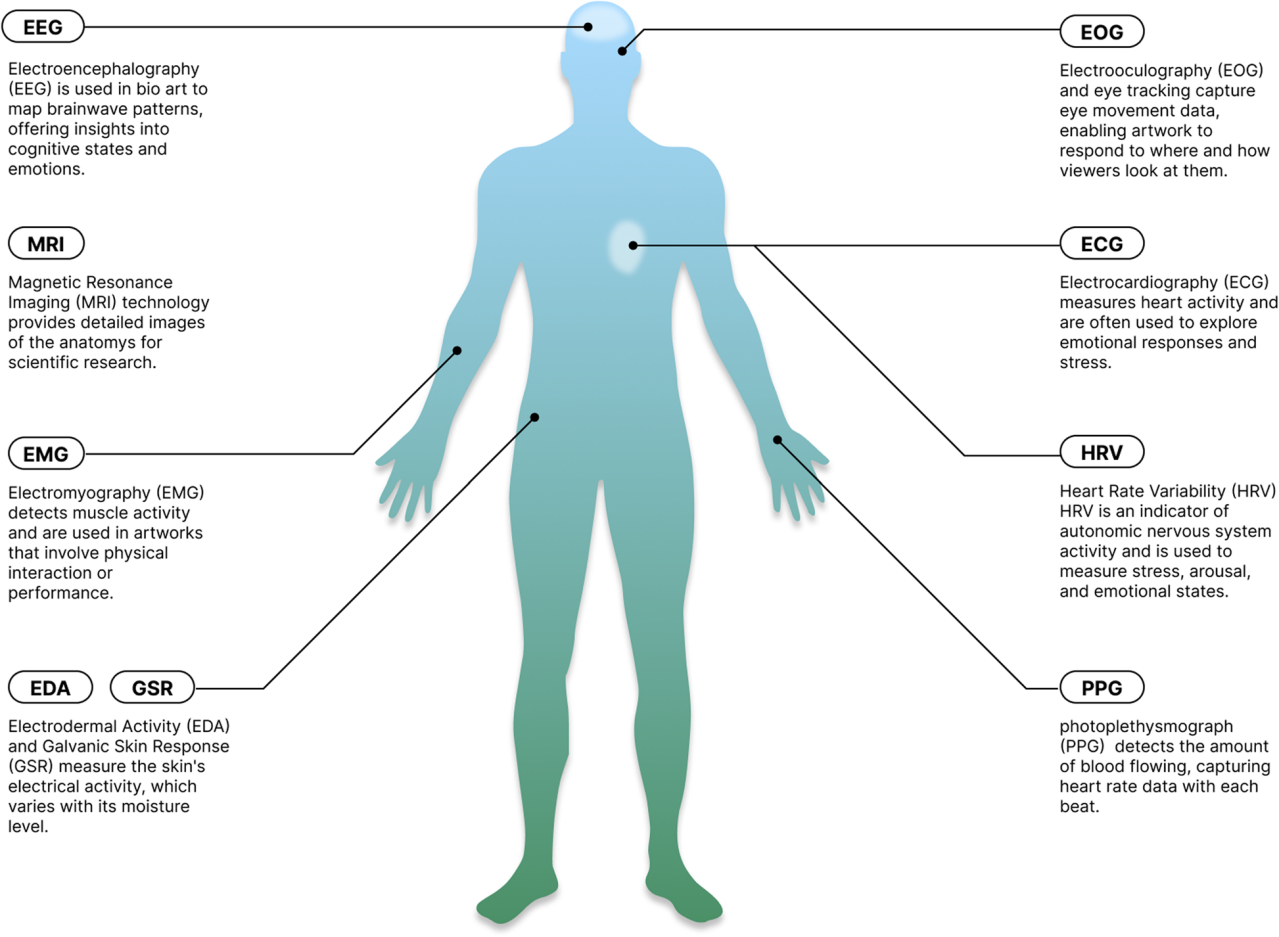
Human body and biosensors

Human biology focuses on the use of human biological information such as structural and functional biodata to explore the themes of augmentation, the inner self, symbiosis, identity, and body politics. Recent artistic explorations have leveraged advances in reconstructing structural data from two-dimensional (2D) to three-dimensional (3D) medical images [32] as well as advancements in processing functional data captured from human bio-activities. These activities include brain, muscle, heart, eye, and skin functions, which are monitored using biosensors such as EEG, magnetic resonance imaging, electromyography, electrocardiography, heart rate variability, electrooculography, electrodermal activity, and galvanic skin response (Fig. 1).

The brain is a complex system in human biology, with dynamic electrochemical activity. Neuroimaging methods, including both structural and functional imaging, offer insights into the brain’s anatomy and functional operations [16]. Structural imaging such as MRI focuses on the physical aspects of the brain, whereas functional imaging such as fMRI and EEG captures active processes across various brain regions.

This survey focuses specifically on artwork utilizing real-time brain wave data. EEG, one of the most common biosensors in BCI artistic expression, is favored for its noninvasiveness, relatively low cost, and high temporal resolution. In this survey, nearly all real-time brainmedia employ EEG as a sensing technology.

### Cybernetics

Developed in the mid-20th century, cybernetics emerged from interdisciplinary dialogue among scientists, including mathematicians, engineers, biologists, and social scientists. It offers a framework for understanding complex systems, emphasizing communication and control through the principles of feedback loops [33].

First-order cybernetics, which was developed in the 1940s, focuses on the study of animals and machines and their control mechanisms through feedback loops [34]. The early brainmedia of the 1960s was influenced by first-order cybernetics, viewed as experiments with controlled machines [3]. In the 1970s, cybernetics evolved to treat systems not as passive entities, but possessing self-awareness and self-regulation [19], leading to a more participatory view in artistic BCI of the interaction between systems and their environments [3]. This evolution led to what Flora Lysen termed “cybernetic ambiguities,” referring to the paradoxical outcomes and interpretations that emerge from cybernetic systems, especially when those systems involve human-machine interfaces, such as those found in EEG-feedback artwork [3].

Cybernetic ambiguities, particularly the concepts of feedback, control, and system behavior, provide insights into the various control methods currently used in EEG feedback systems. These themes are further elaborated on in the later sections of this paper.

### Media ecology

Media ecology, a term coined in the 1960s by Marshall McLuhan, explores the profound effects of media environments on human perception, understanding, and cultural dynamics. Building on concepts from cybernetics, this field examines the intricate interplay between technology, communication media, and the human senses, suggesting that media act as extensions of the human body [17].

McLuhan’s concept that the media extend the human body [35] aligns well with the augmentation facilitated by BCIs. Media ecology examines how communication technologies shape human culture and consciousness. McLuhan famously stated, “the medium is the message,” and argued that “the ‘content’ of any medium is always another medium: for instance, the ‘content’ of writing is speech, just as the written word is the ‘content’ of print, and print is the ‘content’ of the telegraph [35].” Extending this concept, Jess Rowland proposed that the content of speech is thought, illustrating how media theory unfolds the understanding of cognitive processes [36]. BCIs exemplify this phenomenon as they capture brain signals generated by thought and facilitate interaction with computer systems.

Early brainmedia were deeply influenced by media ecological thinking. Gregory Bateson argued that the self should be seen as an expanded mental field that dissolves traditional boundaries between subject and object, mind and body, as well as self and world through ecological communication [29]. Moreover, media ecology helps elucidate how communicative media continually augments people’s perceptions and shapes their understanding of the environment and society. As noted by Claus Eurich, the evolution of media technology has influenced personal identity and collective cultural and social structures [37].

### Countercultural movement

The countercultural movement in the 1960s and the 1970s was partly a response to the technological and media landscapes described by cybernetics and media ecology. Known for its rebellion against prevailing societal norms, the countercultural movement played a crucial role in shaping the development of new media and technological arts. This movement challenged traditional values and introduced radical approaches for human augmentation, communication, and media environments [38]. It also fostered the exploration of inner selves and contributed to the scientific and artistic experimentation of BCI artworks [3].

During this era, countercultural ideologues and visionaries, such as Stewart Brand, embraced new technologies, driven by the belief that rapidly advancing technology significantly influences various aspects of human life, including consciousness and spirituality [39]. Brand emphasized the interconnectedness of humanity and the Earth, proposing that technology emphasizes the interconnectedness of humanity and the Earth [40].

The countercultural movement can be seen as laying the foundational culture that later supported the open-source movement and democratization of technology concepts that align closely with the ethics and experimental spirit found in contemporary brainmedia [41]. By emphasizing personal empowerment and advocating decentralized control, this movement paved the way for a generation of technologists and artists who view technology as a tool for creative expression and social change.

### Brain art, brainmedia, EEG art, BCI art, and neurofeedback art

The terms “brain art,” ‘brainmedia,’ “EEG art,” “BCI art,” “neurofeedback art,” ‘neuroart’ and “artistic BCI” have been developed by various artists, researchers, and scientists over time.

#### Brain art

This term describes a broader category of art that involves brain activity as a creative medium. It encompasses artistic expression facilitated by brain signals through visual, auditory, and other sensory outputs [1].

#### Brainmedia

Coined by Flora Lysen, brainmedia refers to the historical and envisioned integration of brain activity in art, emphasizing socio-technological practices along with cultural imaginaries and visions [3].

#### BCI art

BCI was first used by Jacques Vidal in the 1970s. BCI art refers to the application of BCI technology in the arts, with pioneers such as Nina Sobell and David Rosenboom [3].

#### EEG art

EEG has been used in art since the 1960s, with Alvin Lucier’s “Music for Solo Performer” in 1965 as a notable early example [1].

#### Neurofeedback art

This term, evolving from EEG feedback, covers the art that utilizes neurofeedback, where brain activity is monitored to influence an external system. The concept of neurofeedback in art, interpreted through the lens of Michel Foucault’s “technologies of the self,” began gaining attention in the 1960s [3].

While sometimes used interchangeably with ‘neuroart’ and “artistic BCI,” these terms are rooted in the shared joy and creativity that transcend the quantitative assessment of technology. They are democratic in ambition, demystify technology and facilitate shared experiences. BCI artists use technology to perceive the inner workings of emotions and create affective spaces that transcend personal and social boundaries [1].

## Range of the presented taxonomy

Understanding the concepts of bio art and interactive art helps in situating this survey within a clearer realm of contemporary art. Exploring the historical context of media ecology, the countercultural movement, and cybernetics enhances the understanding and appreciation of brainmedia artwork and helps address critical questions in contemporary media art.

This survey focused exclusively on real-time brainmedia developed in the 21st century, spanning 2000 to 2024. This research indicates that these artworks predominantly use EEG as a sensing device. This work cataloged 60 artworks spanning a broad spectrum of media, including audio art, visual art, audio-visual art, virtual reality (VR), augmented reality (AR), mixed reality, performing art, and installations.

**Fig. 2:**
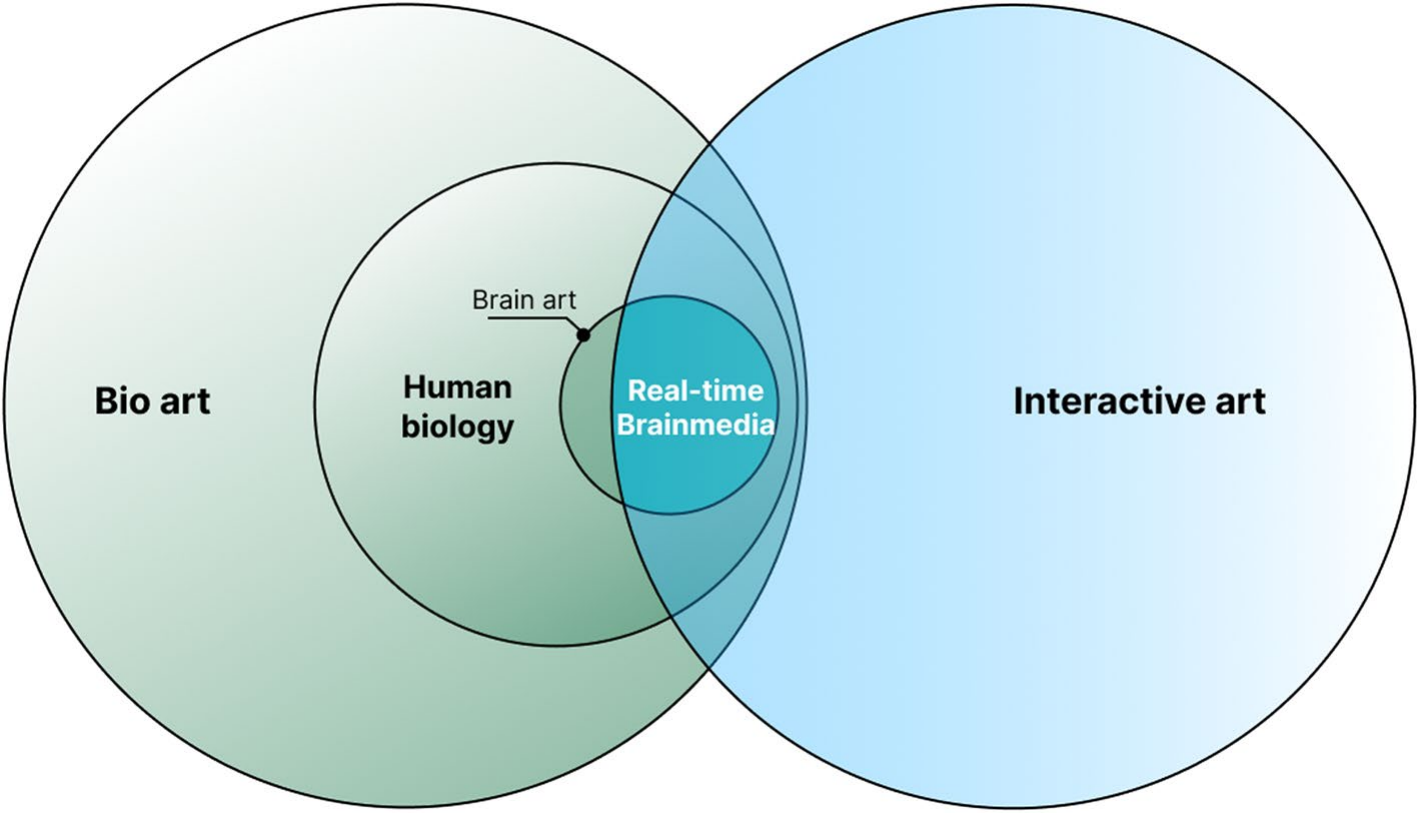
Scope of this survey illustrated by the intersection of the mentioned concepts

By defining a clear and confined scope (Fig. 2), this survey contributes to the academic discourse and serves as a resource for artists, technologists, and theorists interested in the intersections of art, technology, and neuroscience.

## Taxonomy and classification criteria

### Classification criteria

To systematically classify real-time brainmedia as an interactive technological system inspired by Prpa and Pasquier [16], this survey adopted a framework comprising input from participants, processing algorithms, and output media, which were simplified to input, processing, and output components, defined as follows:

1. Input encompasses user roles, interaction modes, and modalities.
2. Processing includes algorithms and remapping methods.
3. Output involves the media format and type of brainmedia.

A large portion of the collected brainmedia artworks did not disclose specific algorithms or remapping methods. Therefore, the processing method was not included as a criterion in the classification here. For the input, the focus was on interaction modes and modalities. Although user roles such as voluntary or involuntary participants, performers, audience members, and collectives present an interesting criterion, some roles overlapped and were closely related to the interaction modes. Thus, user roles were not used as a criterion.

For the output, the brainmedia type that represented the final presentation of the artwork was selected. As contemporary artwork often incorporates multiple media formats media formats were not used as a criterion.

The input-process–output-feedback loop of brainmedia reflects a mapping of cybernetic aesthetics [42] and computation aesthetics [43]. The interaction mode, which is the core of cybernetic aesthetics, is enabled and enhanced by computational techniques that process and respond to human input.

**Fig. 3:**
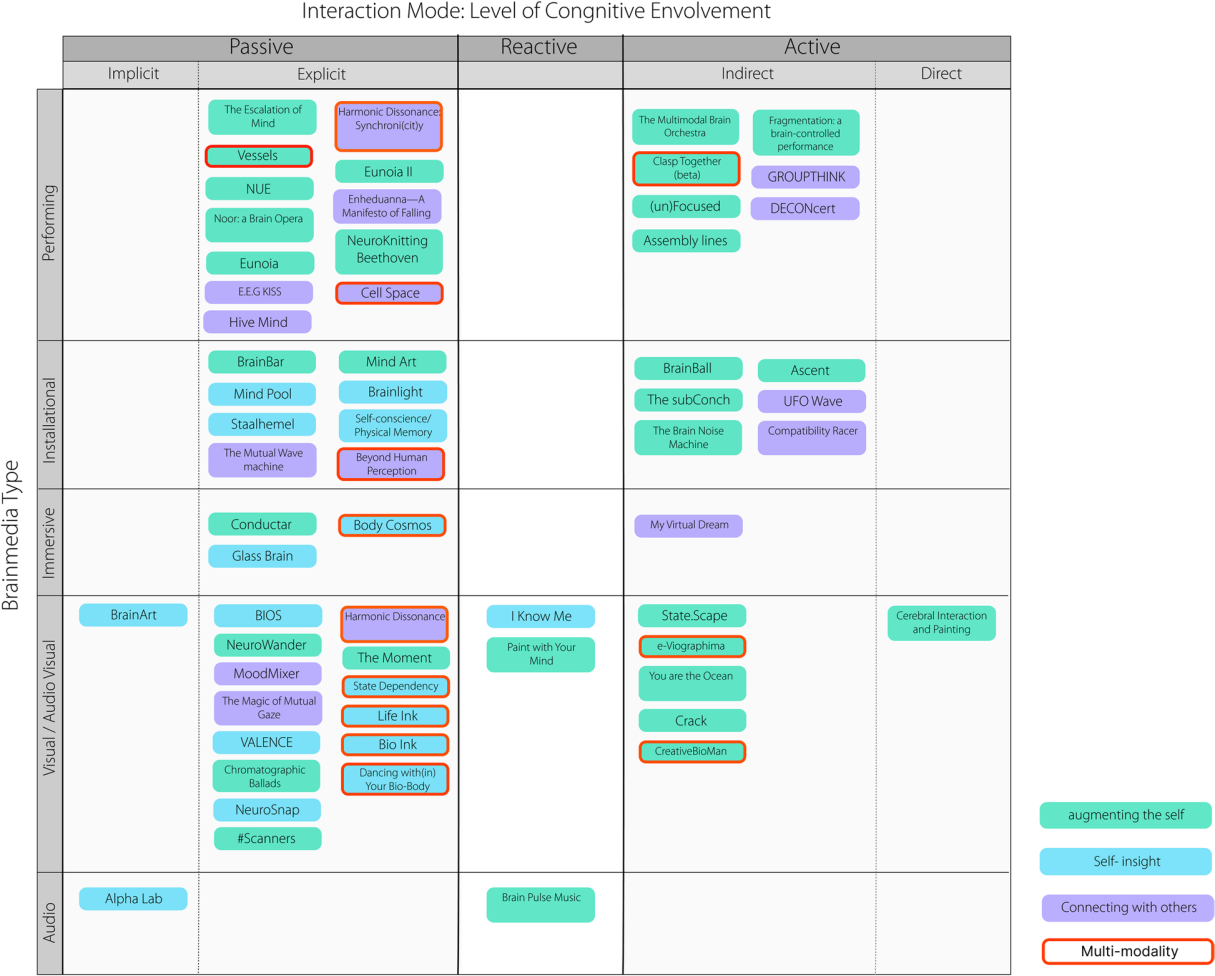
A taxonomy of real-time brainmedia

Here, an XY space was created for classification; the X-axis was divided by interaction modes and the Y-axis by brainmedia type, resulting in a total of 25 subspaces. A thick stroke was used to indicate multimodality, which included brain signals and other bodily signals. The whole landscape unfolded as each artwork was categorized into the appropriate subspace within the XY framework (Fig. 3).

### Interaction mode

The categorization of artwork utilizing BCI varies based on several criteria, primarily the control mode.

In the 1970s, Eaton identified four neurofeedback system control modes: voluntary-involuntary, voluntary-voluntary, involuntary-voluntary, and involuntary-involuntary [1]. This classification addresses both the control modes of the participants and the data mapping methods. Wadeson et al. [44] refined the classification into four participant-based control modes: passive, selective, direct, and collaborative. While the first three categories focus on control modes and mapping methods, the fourth highlights the involvement of multiple participants [16]. Anton classified the control modes as active, reactive, and passive [1], effectively capturing the essence of participant-control methods. Prpa and Pasquier [16] expanded upon this approach, proposing an input, mapping, and output framework that replaces the term ‘control’ with ‘agency’ to better reflect the participatory nature of BCI artworks. Zhang et al. [45] introduced a classification that included active, explicit passive, and implicit passive modes, aligning them with the cybernetic and aesthetic structures in real-time EEG art.

Building on these insights, this work proposes five interaction modes based on varying levels of participant intentionality and interaction with the technological system, in which the term “interaction mode” refers to the concept of interactive art.

#### Implicit passive

Participants are unaware of their role in influencing the artwork or of a more subconscious level of interaction. Given the current limitations of sensing technologies, this mode is deemed to occur when a participant is in a meditative, hypnotic, or sleep state [45].

#### Explicit passive

Participants are aware that their physiological data are being used but do not actively control or alter such data. Their brain waves, which are influenced by their emotional state or focus on other tasks, affect art. Zhang et al. [45] stated that explicit passivity is one of the basic aesthetic structures of performing arts.

#### Reactive

Participants receive stimuli generated by the system or other means. These stimuli are designed to change the subject’s brain signals, which are then captured to affect the output system [1].

#### Indirect active

Participants actively control their emotions, such as attention, relaxation, and pressure, to alter their brain signals. Affective indicators calculated from the brain signals of the subjects are used to control the output parameters.

#### Direct active

Participants actively control their intentions to directly influence the behavior of the output, such as thinking about moving forward to move a ball forward. The direct active mode is less commonly used in BCI artwork because of its technical complexity, training requirements for participants, and reliability challenges.

### Modality

In addition to brain electrical signals, a rich array of biodata exists in human biology, including muscle electrical activity, heart electrical activity, eye voltage differences, and the skin’s electrical conductance. A small portion of brainmedia artwork employs a multi-modality approach to combine brain signals with other biodata, treating the human body as a complex system. Recent artworks go further to incorporate basic and interbody data into brainmedia through motion capture, hand tracking, and collocated AR, manifesting an emerging trend of entanglement of the mind, brain, and body.

### Brainmedia type

This survey categorized brainmedia according to media format, presented into five types: audio, visual/audio-visual, immersive, installation, and performing art.

#### Audio

Audio brainmedia converts brain signals into sound or music. This format was commonly used in the early stages of artistic BCI experiments, beginning with Alvin Lucier in 1965 [4], followed by Richard Teitelbaum [5] and David Rosenboom [6].

#### Visual/audio-visual

This category includes converting brain signals into visual media, such as images, animations, control of movies, or generative art. Although some researchers have differentiated between visual and audio-visual media [16], they are combined here into one category. The earliest example of visual brainmedia dates back to the early 1970s, with Sobell’s Brainwave Drawing Game [46] (Fig. 2). Among the brainmedia artworks collected from 2000 to 2024, nearly half of the entries were classified here as visual or audio-visual.

#### Immersive

Immersive brainmedia involves creating environments that envelop participants with stimuli generated from their brain signals. These include 360-degree projections, VR, and AR setups.

#### Installation

Installation of brainmedia refers to physically based artworks such as kinematic structures, liquids driven by vibration, and environments altered by the brain signals of participants using lights, lasers, and other media.

#### Performing art

Performing brainmedia includes live performances in which brain signals are used to influence the performance or interact with the audience. Brainmedia performance involves two types of potential audience members: active and passive. The brain waves of an active audience member, which can influence the performance, are also captured.

## Objectives when using brainmedia in art

Building on this work’s taxonomy and classification criteria allows further exploration of the impact and goals of brainmedia artworks. These can be broadly grouped into three key objectives: augmenting the self, gaining self-insight, and connecting with others. Each of these objectives highlights the different ways in which brainmedia art can influence participants and viewers, reflecting the interplay between technology and human experience. These categories are deeply influenced by concepts emerging from media ecology, cybernetics, and the countercultural movements of the 1960s and the 1970s, as discussed by Lysen [3].

**Fig. 4:**
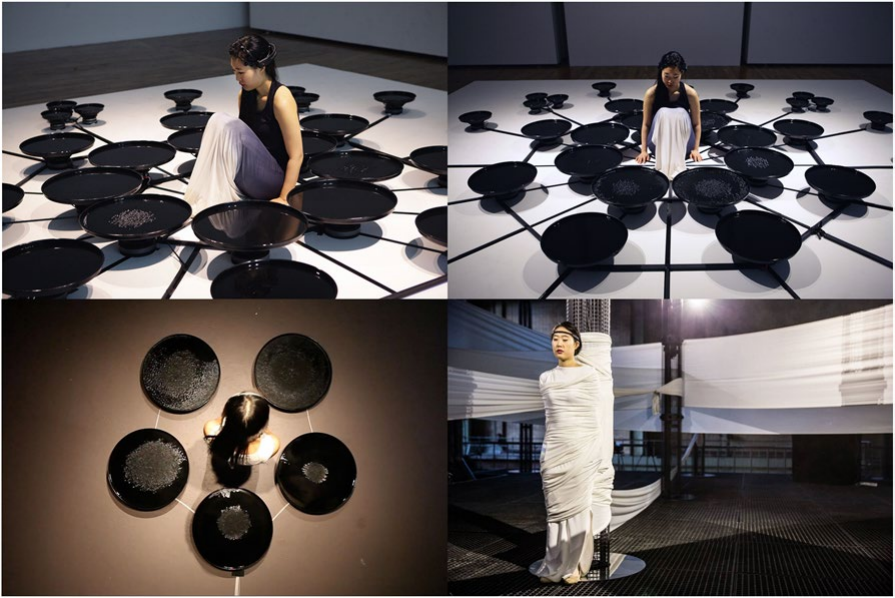
Lisa Park, Eunoia (bottom-left), 2013; Eunoia II (top-left and top-right), 2014; NUE (bottom-right), 2015. With the permission of Lisa Park

All the artworks with different objectives are illustrated by the three node colors in Fig. 4. The remainder of this paper is presented in the order of augmenting the self, self-insight, and connecting with others, followed by a discussion of the historical context and potential future developments.

### Augmenting the self

The notion of augmenting the self through BCI artwork resonates with early cybernetic theories [19] and the idea that media can extend human capabilities [35]. In a historical context, the first EEG-feedback setups gained public attention in the 1960s and the 1970s. Artists and scientists explored the potential of EEG as a control mechanism for machines, marking a significant departure from traditional art forms [3]. Early examples of this artistic exploration as self-augmentation include the work of Edmond Dewan, who experimented with controlling alpha waves [47] and Alvin Lucier’s “Music for Solo Performer,” which utilized EEG feedback to create a sonic composition [4].

Marshall McLuhan’s philosophy, which posits that “media is the extension of the human body [35],” resonates with the use of EEG in BCI art as a form of self- augmentation. Here, the EEG is not just a tool for measuring brain activity; it becomes an extension of the self, allowing artists and participants to interact with technology based on their inner states. This interaction extends an individual’s perception of their own cognitive processes and represents a form of self-augmentation through artistic expression.

This concept continues to be explored in the 21st century, where the brain’s electrical activity, measured by EEG, acts as a brainmedia self-augmentation in artistic expression.

#### Audio brainmedia

In the 21st century, audio brainmedia is relatively rare. Notable examples include “Brain Pulse Music,” an avant-garde album by Masaki Batoh that blends traditional Japanese instruments with innovative compositions controlled by brain waves [16]. Using a custom-built device that converts EEG signals into music through a reactive interaction mode, Batoh’s project pushed the boundaries between music and technology, exploring new realms of auditory expression.

#### Visual/audio-visual brainmedia

**Fig. 5:**
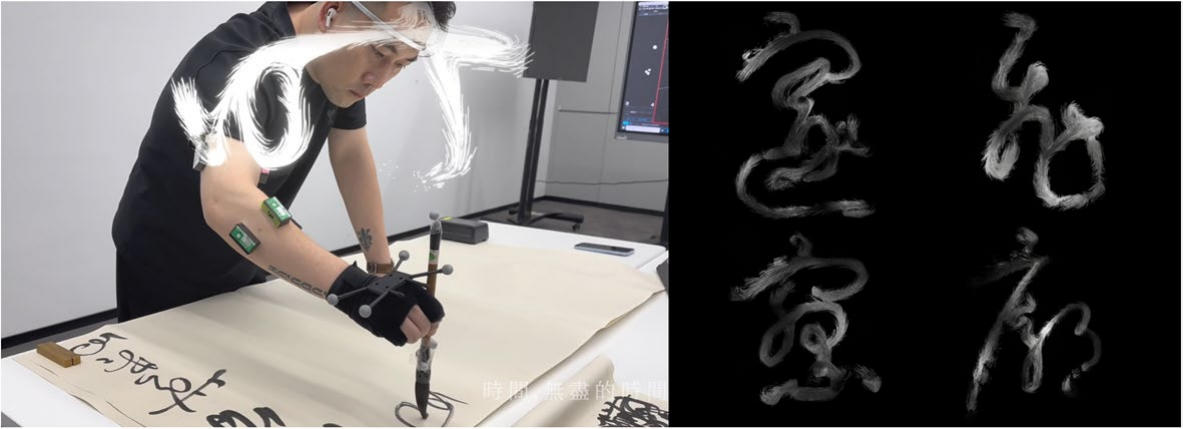
Lin et al., Bio-Ink, 2023

BCI is utilized as a control method to augment the self in games, exhibitions, films, and creative tools, thereby demonstrating the expansion of interactive storytelling. For instance, NeuroWander enables players to passively navigate a virtual world via their brain signals [48]. Ursula Damm’s installation, Chromatographic Ballads utilizes participants’ passive explicit EEG data to interact with Neurovision software, manipulating live video feeds to alter their audio-visual perception [49]. State.scape (2014) animates a flock of birds based on users’ active affective states, influencing behaviors such as flight speed and path in real time [50]. You are Ocean allows participants to control natural phenomena, such as ocean waves and weather patterns, with their active affective states [51] (Fig. 5). The Crack, an EEG-based artwork, enables participants to create visual cracks in a concrete installation through active concentration, combining environmental activism with interactive art [52]. These four artworks resonate with the notion of media ecology [17], highlighting the influence of internal states on external environments and suggesting the impact of human intentions on the environment and the natural world.

BCI control has also been applied in cinematic experiences. Scanners dynamically responds to the physiological data of its viewers, specifically their passive explicit brain activity and blinking patterns captured through an EEG device [53]. The film consists of four parallel channels of footage that change according to the viewer’s level of attention, meditation, and blink rate. This research-led design reveals a 2D design space that maps the balance between voluntary control and user awareness of that control, thereby offering new insights into interactive entertainment design. Richard Ramchurn, one of the authors of Scanners, continued to develop another BCI interactive sci-fi film titled The MOMENT [54]. The film employs a Neurosky headset to monitor the EEG brain waves of audience members, allowing the movie’s editing, sound mix, and narrative to respond dynamically to the viewer’s level of attention and meditation. These two projects push the boundaries of interactive storytelling by making the audience’s cognitive state part of the narrative process.

BCI can also serve as a creative tool when combined with gestures or other biometric data for artistic exploration. E-Viographima integrates EEG and hand-tracking technologies to create visual art based on artists’ active brain activity and hand movement [55]. CreativeBioMan utilizes EEG along with a wearable expressive robot that collects brain wave, noise, real-time creation data, and historical data of the artist [56]. These data are then processed by AI algorithms to generate an artwork that is stylistically consistent and reflects the current emotional state of the artist. This type of multi-modality brainmedia entangling the brain, mind, and body is a trend in recent artworks, illustrating the integration of multimodal data and personalized artistic expression.

#### Immersive brainmedia

Combining brainmedia with spatial computing technologies, such as AR, creates a virtual layer on the physical world through the extension of the inner self. Conductar: Moogfest is an AR artwork that transforms the city of Asheville into an interactive, audio-visual landscape for Moogfest attendees [57]. This immersive experience transforms the city into a dynamic canvas for exploration and creation.

#### Installation brainmedia

The Interactive Institute, founded in 1998, conducts research on art, design, and interactive media. In the early 2000s, they developed brainmedia art. One of the most notable, Brainball, garnered significant media attention and received an honorary mention at Ars Electronica 2000. Described as an “anti-game,” Brainball subverts traditional gaming paradigms by rewarding active relaxation over excitement [58]. This installation utilizes an indirect active interaction mode in which two players, seated opposite each other with EEG headbands, influence a small steel ball on a clear plastic surface. The ball moves away from the player who is the most relaxed toward the other player. By making the objective to generate alpha and theta brain waves, which are associated with calm and relaxation, Brainball critiques the competitive nature of conventional gaming. Brainbar is another brainmedia artwork developed by the Interactive Institute in 2001 [59]. Brainbar integrates BCIs into everyday life using brain waves to mix drinks that reflect the user’s emotional state, demonstrating BCIs’ potential to provide personalized experiences through personal inner states. In addition to the brainmedia installations created by the Interactive Institute, other installations, such as subConch [16], ASCENT [60], and The Brain Noise Machine [61], use EEG technology to create immersive experiences that challenge the traditional notions of digital interactions.

#### Performing brainmedia

Performing art integrating BCI has a long history, traceable back to Alvin Lucier’s “Music for Solo Performer” in 1965 [1]. Performing brainmedia showcases the expansive potential of BCI technology in live performances, transforming artists’ intentions and audience expectations. Contemporary artists such as Lisa Park have used BCI in performances to create dynamic, responsive environments (Fig. 4). In 2013, she created a performance titled Eunoia, which means “beautiful thinking” [62]. She used an EEG to convert her mental state into auditory vibrations that influence water surfaces, thereby highlighting the connection between mental activity and external expressions. She enhanced Eunoia in 2014 by integrating 48 speakers, arranged in a layout inspired by an Asian Buddhist symbol meaning ‘balance’ [63]. In 2015, Park continued to explore performing brainmedia with an artwork called NUE inspired by the silkworm’s life cycle [64]. Wearing a 150-meter white dress, Park transformed a physical space into a responsive environment shaped by her brain waves. A specially designed mobile application captures various brain wave patterns (concentration, mellow state, alpha, beta, delta, theta, and gamma) from an EEG headset via Bluetooth and transmits Open Sound Control data messages to the 4DSOUND system. This setup allows a soundscape shaped by the artist’s brain data to unfold, immersing the audience within the cocoon’s enveloping white fabric. This dynamic interaction between the artist’s mental state and the environment illustrates the capacity of BCIs to augment artistic self-expression and create immersive, personalized artistic experiences. Ellen Pearlman’s ‘Noor’ uses EEG to create an interactive brain opera, engaging with the themes of surveillance and privacy [65]. Other audio- or visual-based performing brainmedia include The Multimodal Brain Orchestra, which allows performers to generate and control music directly through brain signals, demonstrating a new form of collective artistic creation [66]. The Escalation of Mind melds brain wave interaction with audiovisual synthesis to augment traditional theater, using live EEG data to dynamically influence performance [49]. Clasp Together (beta) redefines musical expression by converting performers’ physical movements and cognitive states into sound [67]. Grace Leslie’s Vessels integrates EEG, Electrodermal Activity, and ECG with musical improvisation to explore the interplay between physiological signals and artistic expression [68].

The Seoul version of NeuroKnitting Beethoven is a performing brainmedia that used brain waves from an audience member (a Buddhist monk) onsite, rather than from the performer (pianist), to control a knitting machine during live musical performances [69]. This approach transforms a passive audience into an active one whose cognitive states translate into textile patterns.

### Self-insight

The development of BCI transitioned from using brain-controlled machines to exploring the inner self in early experiments [3] and was deeply influenced by the countercultural movement. This shift was partly driven by the emerging connection between recorded alpha brain waves and states often associated with meditation and spirituality, with EEG feedback being seen as a tool for providing deeper insights into the inner self [70].

Another key concept is Foucault’s “technologies of the self,” which involves understanding the self as an entity that can be deeply explored, transformed, and controlled using various methods and technologies [71]. Such technologies include flotation tanks, psychedelic drugs, stroboscopic devices for inducing visual stimuli, and mutable architectures that adapt to inhabitants [3].

The enthusiasm for EEG feedback in the late 1960s was driven by its potential to offer new insights into one’s mental and emotional processes by observing and modifying one’s brain waves, particularly the alpha waves associated with relaxation. This introspective approach reflects a broader cultural shift toward valuing personal discovery and inner exploration [3].

The notion of “technologies of the self” and the pursuit of self-insight are deeply rooted in the historical context of artistic BCI and continues to influence brainmedia into the 21st century.

#### Audio brainmedia

Alpha Lab/Theta Lab is an experimental art research project that explores the ‘self’ in the intersections between consciousness and sub-consciousness, intertwining neurofeedback with participatory art and electronic music, featured at ISEA2013 [16]. The Alpha Lab/Theta Lab harnesses the training of alpha and theta brain waves through neurofeedback to guide participants into a deep, hypnagogic state between wakefulness and dream.

#### Visual/audio-visual brainmedia

Visual/audio-visual brainmedia provides an artistic visualization technique for participants to explore their inner state. I Know Me is an artwork that utilizes EEG to reflect participants’ mental states using dynamic visuals based on Lissajous curves [72]. This project captures brain wave activity, specifically alpha and beta waves, to depict emotions such as anger or sadness in real time, enabling participants to gain deep insight into their own mental conditions and potentially learn to modulate their emotional responses, thus enhancing self-awareness and emotional understanding. Brain Art is another project that delves into the subconscious and unconscious selves by transforming sleep EEG signals into unique, global abstract images [73]. These images provide a visual interpretation of the nuanced structures of sleep, aiming to encode nighttime sleep EEG data into aesthetically pleasing visual sequences that articulate the complex dynamics and cycles of sleep and dream processes. VALENCE is an interactive visualization for live brain wave monitoring using a wireless EEG headset to monitor a player’s alpha waves (an indicator of relaxation) and valence (an indicator of emotion or arousal) [74]. NeuroSnap redefines the facial filters on social media by integrating a BCI to adjust filters to reflect the user’s affective states derived from EEG data, visually reflecting the user’s level of relaxation and alertness in communication [75].

As brainmedia increasingly incorporates the multimodality of biodata, it extends the understanding of “technologies of the self” [71], providing more comprehensive insights into human biology. Life Ink by Ars Electronica Futurelab in collaboration with Wacom, transforms human brain waves and body signals into visual art. Customized wearable gears capture movements and sweat gland activity to create a dynamic stream of 3D “Life Ink” [76]. This ink visually manifests the wearer’s emotional and creative states through vibrant displays of color, light, and form, offering a novel way to visualize and interpret human creativity. Life Ink has been showcased by various creatives, including pianist Maki Namekawa, in her performance at the Ars Electronica Festival.

Rem Rungu Lin is a digital artist and researcher who investigates the intersections of bio art, mixed reality, and human-computer interaction. His research and artwork create a series of interdisciplinary, multimodal brainmedia artworks that entangle the mind, brain, and body. One such piece, Bio-Ink, is a generative artwork inspired by the Chinese traditional concept of ‘Qi,’ creating dynamic sculptures of cursive calligraphy through a particle system that incorporates EEG, EMG, and motion data [77] (Fig. 5). The motion data capture the shape of the calligraphy, whereas the biometric data influence various parameters such as the size, brightness, and noise of the particles. The integration of biofeedback mechanisms enriches the artwork by revealing the spatiotemporal nuances of an artist’s creative process. Lin further explored the historical context of ‘Qi,’ connecting this ancient Chinese philosophy—which encompasses concepts like the life force (“Yuan Qi”) that connects all things, the dual dynamics of Yin and Yang, vital essence (‘Jing’), energy (‘Qi’), and spirit (‘Shen’)—with a contemporary understanding of human biology [77]. This profound connection resonates with Foucault’s “technologies of the self” [71], suggesting that both ancient wisdom and modern technology explore similar themes of interconnectedness and self-awareness.

**Fig. 6:**
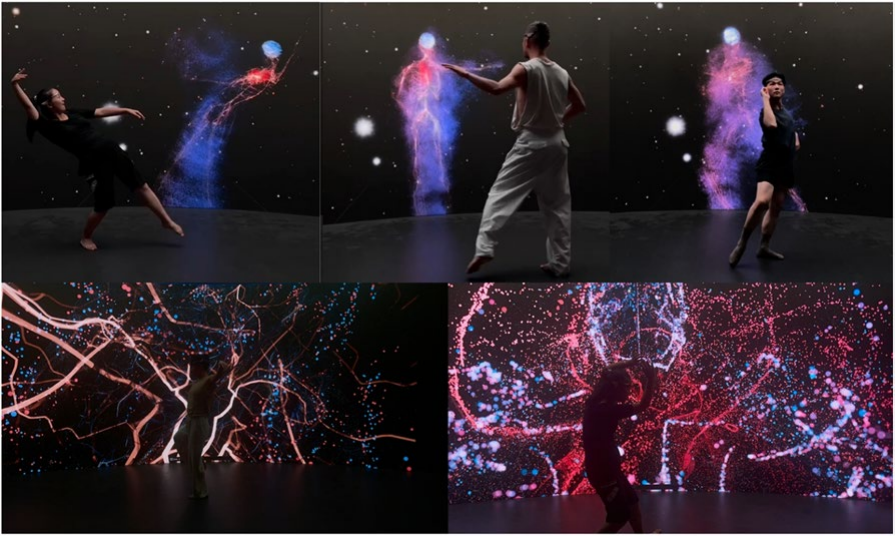
Lin et al., Dancing with(in) Your Bio-Body, 2024

Rem Rungu Lin developed another artwork titled Dancing with(in) Your Bio-Body, which stands at the intersection of dance, technology, and artistic BCI [78] (Fig. 6). This artwork introduces the ‘bio-body,’ which is a dynamic digital representation of a dancer’s internal state, generated in real time using EEG, heart rate, and motion capture. The system offers two interactive experiences: one immersing dancers in their internal physiological states, and the other creating a bio-digital mirror for enhanced performance. This artwork transcends traditional neurofeedback by incorporating the concept of embodied cognition and exploring new dimensions of the interplay between technology and artistic expression.

#### Immersive brainmedia

The combination of brainmedia with AR and VR enhances self-insight by immersing users in environments that respond to and are modified by their inner state. Glass Brain, developed by the Intheon team, is the world’s first interactive, real-time, high-resolution visualization of active human brain activity and connectivity optimized for VR environments [79]. This pioneering project transforms neurological data into a visually immersive VR experience, offering unprecedented insights into brain function.

**Fig. 7:**
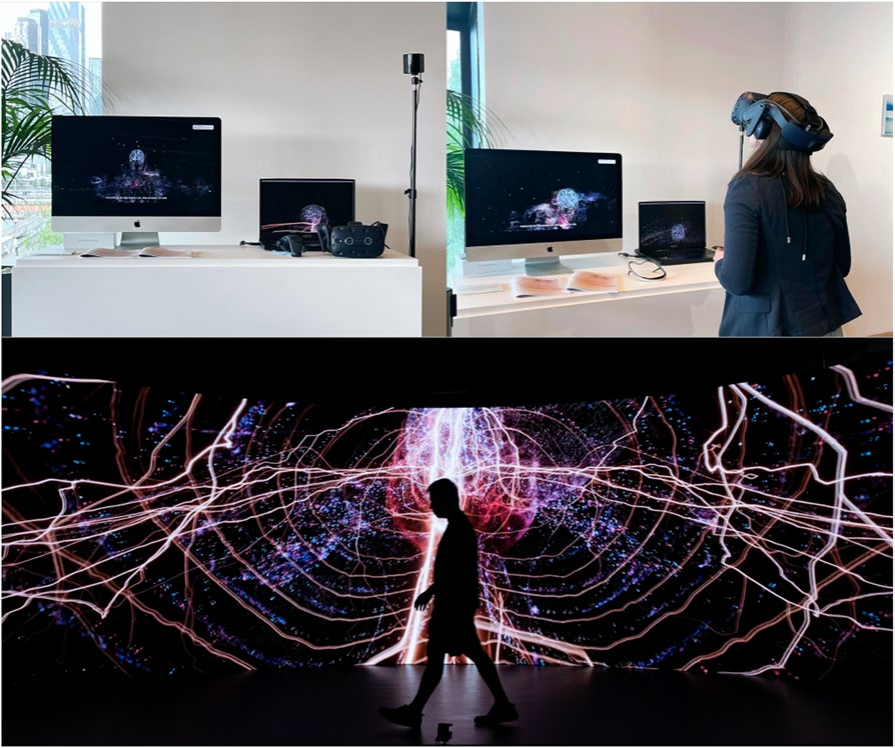
Lin et al., Body Cosmos, 2023

Body Cosmos by Lin et al. [80] is a VR artwork that fosters a symbiotic relationship between the human body and a simulated cosmic environment (Fig. 7). Utilizing volumetric rendering and particle systems, this artwork merges human structural imaging (DICOM) with real-time functional imaging (EEG) and heart rate data to create a simulated blend of the human anatomy and nebulae. Body Cosmos invites participants to travel inside their bodies while simultaneously connecting them to the universe. This immersive experience deepens the users’ understanding of themselves and redefines traditional perceptions of the human body in relation to the cosmos. It offers a unique perspective on selfhood, embodiment, and identity, thus challenging and expanding the conventional boundaries of interactive and immersive brainmedia.

#### Installation brainmedia

Installation brainmedia fosters self-insight by materializing brain data into tangible interactive art installations, thereby enriching the fields of both neuroscience and interactive art. These installations render complex brain processes accessible to the public.

Mind Pool is an installation designed to foster deep self-reflection by providing real-time physical interaction with a participant’s brain activity [81]. This system features a pool of magnetically reactive liquids embedded on a tabletop, where changes in brain activity are translated into movements within the liquids, offering unique sensory reflections of cognitive states. Staalhemel comprises 80 kinetic ceiling tiles that sonically map the topography of the brain [44]. Visitors wear a wireless EEG interface that translates their brain waves into acoustic taps on the steel tiles, creating a tangible auditory space that mirrors the intangible flow of thoughts. Self-conscience/Physical Memory is another robotic ceiling sculpture consisting of motorized and illuminated acrylic tiles that transform architectural space in response to EEG data [82]. The tiles’ movements and color changes respond to the participants’ brain activity, allowing the piece to act as a physical representation of past experiences and memories, thus making the intangible aspects of identity and consciousness tangible. Brain Light is an interactive sculpture that illuminates the hidden dynamics of people’s minds [83]. This artwork uses an EEG headset to detect live neural activity, which is then translated into a light display within the sculpture. Theta waves are represented as green light, alpha waves as blue light, and beta waves as red light, creating a real-time visual representation of the viewer’s mental state. Brain Light has been used to explore the inner states of performers and audiences during performances, thereby enhancing the communal experience of art and introspection.

### Connecting with others

Emerging around 1970, the concept of the ‘self’ developed from exploring the inner self to envisioning the self as “connected with—or plugged into—broader circuits or systems” [3]. This shift was catalyzed by new interest in real-time EEG technologies, resonating with the radical ideas of the countercultural movement [18] and media ecology [17].

The emergence of the “circuited self,” a term introduced by Lysen [3], models the world as a “total environment.” Early explorations of this concept can be traced back to the 1970s, notably through the Ecology of the Skin by Rosenboom in 1970 [6] and the Alpha Garden by Jacqueline Humbert in 1973 [84]. Rosenboom’s work contributes to an expanding understanding of ‘ecology’ [29], fostering the concept of ‘synchrony’ [3].

The concept of ‘synchrony,’ which focuses on artistic expression and human connectedness, is considered the temporal alignment or mutual adaptation of brain rhythms. This synchronization can occur within the brain (as in neural oscillations), between individuals (as in social interactions), or between individuals and their environment [85].

These ideas continue to profoundly influence the exploration of multi-brain BCIs and inspire artists to explore new paradigms of current human-machine-space symbiosis.

#### Visual/audio-visual brainmedia

**Fig. 8:**
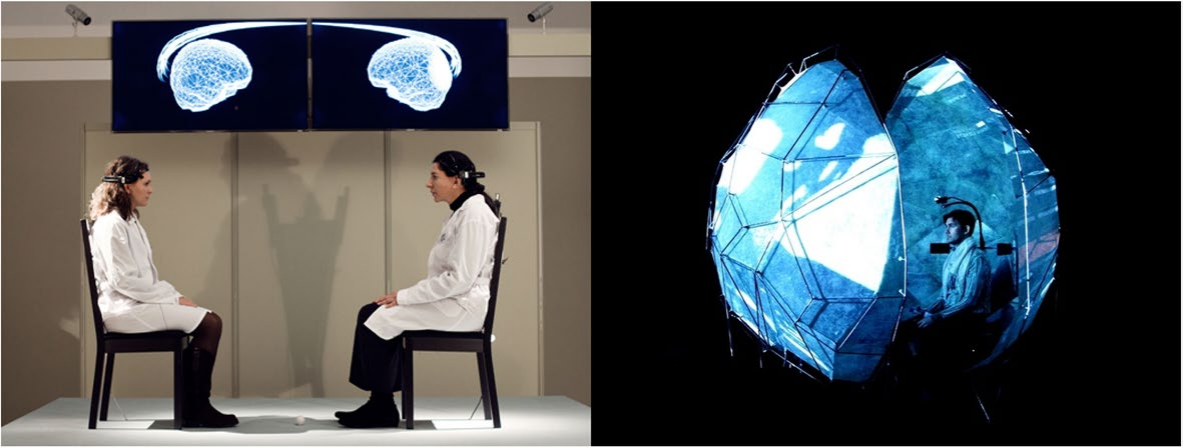
Abramovic et al., Measuring the Magic of Mutual Gaze (left), 2011; Oostrik et al., Mutual Wave machine (right), 2013; Permissions granted

Suzanne Dikker’s work blends cognitive neuroscience, performance art, and education. She adopted a ‘crowdsourcing’ approach in neuroscience to explore the neural underpinnings of dynamic human social interactions [86]. Among her projects is the Harmonic Dissonance collective, which includes visual/audio-visual, installation, and performing brainmedia. The Magic of Mutual Gaze is an interactive artwork that explores the potential of brain wave synchronization to foster human connectedness [85] (Fig. 8). In this installation, two participants engage in a 30-minute mutual gaze while their EEG signals are captured and is visualized in real time. Synchronization is dynamically illustrated through a lightning animation that connects two model brains. Harmonic Dissonance offers an immersive experience in which groups of visitors interact with an ever-evolving social network visualized through light patterns generated based on their brain wave and motion synchrony [85].

Additionally, other visual/audio-visual brainmedia, such as MoodMixer, explore the potential of EEG technology in a musical context, enabling participants to navigate and alter musical soundscapes through their cognitive state [87]. EEG KISS uses two BCIs to mediate social touch in public spaces, transforming familiar sensory interactions, such as seeing, hearing, touching, and moving, into unfamiliar sensory syntheses linked to data visualization and audification of brain activity [88].

#### Immersive brainmedia

Emerging technologies such as AR and VR enhance connectivity by providing immersive environments that extend embodiment and identity and introduce new layers of perception to bodily interaction. The application of AR and VR in exploring human connections demonstrates significant potential, although the catalog produced here shows a notable lack of artwork in this category.

 Virtual Dream is a large-scale dome projection mapping experience that engaged 523 participants in a collective computer game aimed at manipulating mental states of relaxation and concentration through neurofeedback [89]. Despite being categorized as an immersive brainmedia, it does not employ typical AR or VR technologies. Cell Space represents the latest advancement in performing brainmedia, utilizing AR to create a new layer of perception, which is explored in greater detail in subsequent section [90].

#### Installation brainmedia

Wave UFO, created by Mariko Mori, is a renowned installation that merges art, architecture, science, and interactive technology to explore the connections between human consciousness and the cosmos [91]. The brain waves of the participants are monitored and visually projected as colorful dynamic cells onto the ceiling. These visuals, which reflect states such as relaxation and alertness, draw on Buddhist concepts of interconnectedness and life cycles, offering self-awareness and a universal connection.

The DECONcert series integrates brain waves with environmental elements to explore collective consciousness within a performative setting [92]. Using water as both a medium and metaphor, DECONcert delves into the dissolution of individuality in digital and neural networks, prompting reflections on the interconnected nature of modern identities.

Compatibility Racer and The Mutual Wave Machine, which are installations by Suzanne Dikker, explore interpersonal synchrony through BCIs. Compatibility Racer is a competitive, interactive brain-robotics installation in which participants’ brain wave synchronization directly affects the speed of a cart on a track, transforming brain wave synchronization into an element of gameplay [93]. The Mutual Wave Machine places two participants inside an intimate capsule surrounded by an audiovisual environment that dynamically responds to their combined brain activity [85] (Fig. 8). Synchronization increases the vividness and coherence of patterns, whereas a lack of synchronization leads to chaotic, darker visuals and sounds.

Beyond Human Perception extends the concept of connection beyond human-to-human to include human-plant responses to live music [94]. Brain activity from the participants, captured via EEG, and plant electrical oscillations detected by a custom sensor are visualized in real time as moving spheres within a torus shape. This setting provides a perspective on cross-species communication and responses to environmental changes.

#### Performing brainmedia

Enheduanna—A Manifesto of Falling is a live brainmedia cinema performance that utilizes multi-brain BCIs to enable real-time interaction among participants, including performers and audience members [95]. Using a passive EEG-based BCI system, it captures and analyzes EEG data from seven participants across three events to examine the cognitive and emotional connections between the performer and the audience. Hive Mind explores cooperative cognition through an on-stage interaction in which two performers communicate using brain-generated light and sound pulses that synchronize the audience’s brain oscillations, creating an immersive experience of collective consciousness [96]. Harmonic Dissonance: Synchroni(cit)y a dance performance by Suzanne Dikker and her team, investigates the concept of “meaningful coincidences” as described by Jung [85]. It explores the potential unity between and within individuals, challenging the traditional notions of mind-body duality and the ideal of togetherness.

GROUPTHINK extends the dynamic interplay between live performers and their audiences to online live-streaming events, aiming to redefine ‘liveness’ in performance. Traditional live performances thrive on physiological feedback mechanisms such as clapping and dancing, which are diminished in online settings, limiting interaction with text and icon-based feedback. GROUPTHINK envisions a future “Internet of Neurons” where live performance interactivity is enhanced by capturing and conveying the physiological reactions of remote audiences [97].

**Fig. 9:**
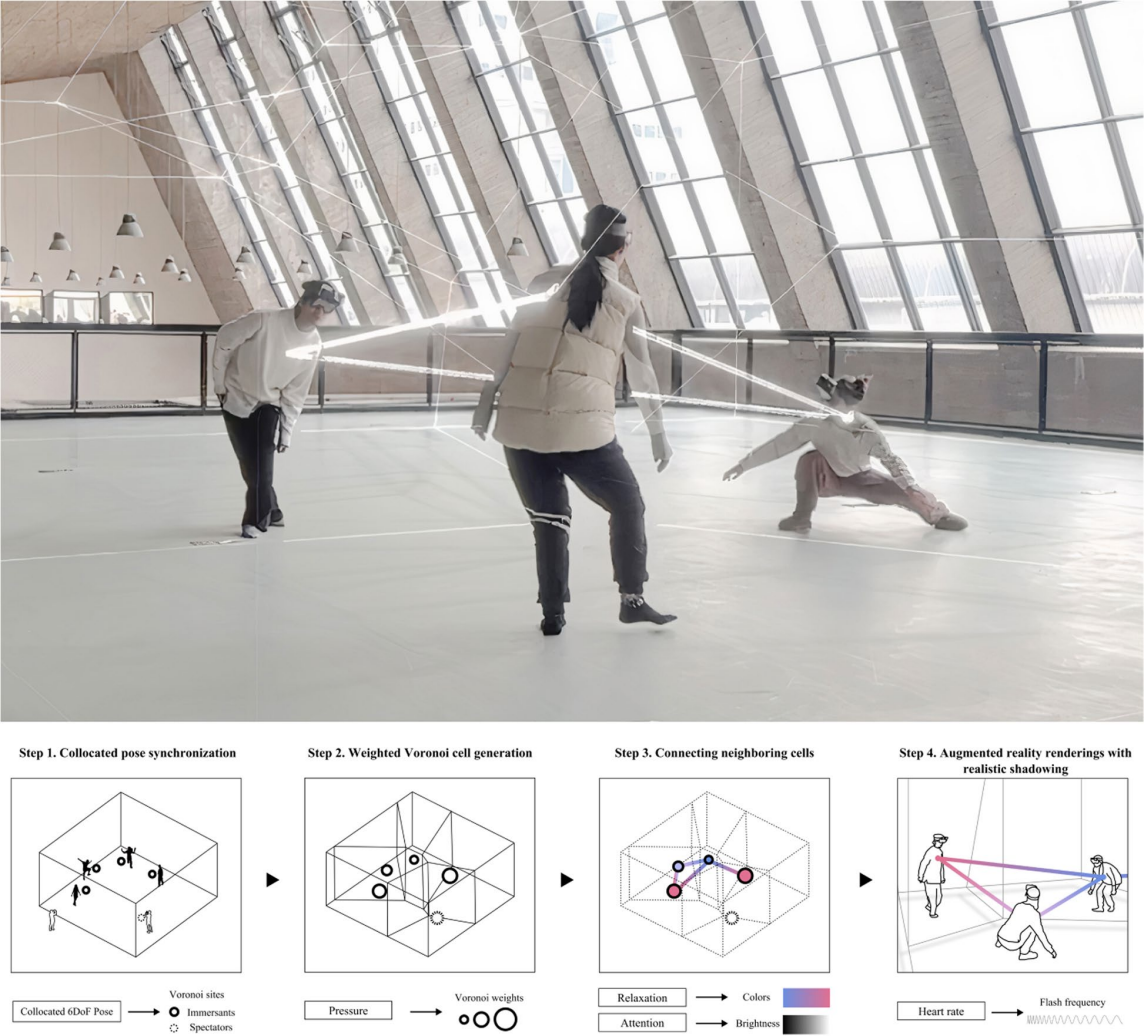
Lin et al., Cell Space (top), 2024; The Neurofeedback System of Cell Space (bottom), 2024

Cell Space, recently developed by Lin et al. [90], integrates collocated AR, neurofeedback, and contact improvisation (Fig. 9). Influenced by McLuhan’s media ecology [17] and the Art Manifesto’s vision of “we becoming the Media [98],” this project envisions humans as cells within an evolving organic spatial entity, resonating profoundly with the concept of the “circuit self”—the self being integrated within broader circuits or systems [18]. Performing brainmedia advances the concept of intercorporeality, moving beyond mere physical interaction to a “shared bio-digital domain” [90]. Cell Space also introduces a new paradigm in the performing arts by applying AR and neurofeedback in contact improvisation, creating a new layer of perception and enriching intercorporeal interactions. This project reinforces the individual and collaborative intentionality of its participants and acts as a reflective mirror of the potential future of humans’ increasingly interconnected existence.

## Discussion

The development of brainmedia reflects people’s evolving understanding of the mind, brain, and body, transitioning from brain control to envisioning the self as integrated into broader circuits or systems [3]. This progression is deeply rooted in the historical context and continues to influence contemporary brainmedia artworks. The notion of brainmedia will continue to evolve in response to advancements in machine learning, augmented/virtual/mixed reality, multi-modality processing, human-computer interaction, and sensing technologies. This section explores emerging trends that potentially will shape the future of brainmedia.

### Expansion of interactive storytelling

BCI technology in games, exhibitions, and cinematic experiences transforms traditional narratives by making the audience’s cognitive and emotional states dynamic elements of the story. The use of EEG in creative tools allows artists or audiences to infuse their personal states into the artwork, emphasizing personalized expression. Combining EEG with other biometric data (e.g., heart rate and muscle tension) creates richer and more immersive experiences. This interaction introduces a new dimension to storytelling, in which the narrative evolves based on real-time responses, creating a deeply personalized and engaging experience.

### Raising awareness by augmenting the audience

Brainmedia is being used to engage with environmental themes, demonstrating the potential of art to influence public perception and activism. By integrating BCI technology with environmental art, artists can create powerful and impactful works that raise awareness and inspire action on critical issues such as climate change and species extinction.

### From neurofeedback to multimodal biofeedback

Brainmedia has traditionally focused primarily on neurofeedback. Although early experiments such as Ecology of the Skin [6] integrated EEG with other biodata, it was not a feedback mechanism and was very rudimentary. The trend increasingly points toward incorporating multimodal biofeedback mechanisms that integrate neural physiological responses such as heart rate, muscle tension, and galvanic skin response. This holistic approach allows artists to create complex and responsive environments that mirror human experiences more accurately, providing a deeper understanding of human innerscapes and creativity.

### AR and VR for self-exploration

Combining AR and VR with brainmedia allows users to immerse themselves in virtual environments through their brain waves. These immersive environments respond to users’ inner states, enhancing self-insight and broadening the scope of interactive experiences. AR and VR technologies provide a platform for exploring the interplay between the mind, body, and virtual space, thereby creating a rich and multifaceted experience.

### Entanglement of the brain, mind, and body

The exploration of neurofeedback in artistic contexts is advancing toward the concept of embodied cognition, acknowledging that cognitive processes emerge not only from brain activity but also from bodily interactions with others and the environment [99]. This understanding can be further extended by integrating brainmedia with collocated AR technology. AR introduces a new layer of perception and connection, establishing the tangible presence of space and relationships through people’s bodies. This technology bridges the gap between individual subjective experiences and collective awareness, deepening the understanding of the entanglement of the brain, mind, and body in an increasingly mediating world.

### Cross-species communication through artistic BCI

As global warming intensifies and species extinction escalates, research and artistic endeavors are shifting from anthropocentrism to ecocentrism [100]. An emerging trend in artistic BCIs is facilitating communication across species and exploring non-human consciousness and its interactions with human cognition through art. By integrating the neural activities of various species, artists can create works that highlight the interconnectedness of all life forms, modeling the world as a “total environment” [3].

## Conclusions

This survey traced the evolution of BCIs within media art, illustrating the transition from using brain-controlled machines to exploring the inner self and envisioning the self as “connected with—or plugged into—broader circuits or systems” [3]. By addressing the historical context, this work categorized brainmedia into themes of “augmenting the self,” “self-insight,” and “connecting with others.” Through an examination of artworks that utilize real-time brain signals, it showed how BCIs expand artistic expression, delving into profound questions about human perception and consciousness. These explorations challenge the definitions of brainmedia art and engage in cultural, ethical, and philosophical discussions on the nature of consciousness and interconnectedness.

Technological advancements such as mixed reality, machine learning, and multi-modality processing continue to evolve; they increasingly dissolve the boundaries between individual and collective experiences as well as between organic and synthetic realities.

Looking forward, the intertwined world of the brain, mind, and body will continue to push artistic practices and cultural discourse toward the role of technology in society. This survey sets the stage for future explorations that will continue to redefine communication between humans, machines, and the environment.

## Data Availability

Not applicable.

## Abbreviations

BCI: Brain-computer interface
2D: Two-dimensional
3D: Three-dimensional
EEG: Electroencephalography
VR: Virtual reality
AR: Augmented reality
